# Inflammatory Modulation of Toll-like Receptors in Periodontal Ligament Stem Cells: Implications for Periodontal Therapy

**DOI:** 10.3390/cells14060432

**Published:** 2025-03-13

**Authors:** Mohamed Mekhemar, Immo Terheyden, Christof Dörfer, Karim Fawzy El-Sayed

**Affiliations:** 1 Clinic for Conservative Dentistry and Periodontology, School of Dental Medicine, Christian-Albrecht’s University, 24105 Kiel, Germany; stu201748@mail.uni-kiel.de (I.T.); doerfer@konspar.uni-kiel.de (C.D.);; 2 Oral Medicine and Periodontology Department, Faculty of Oral and Dental Medicine, Cairo University, Giza 12613, Egypt; 3 Stem Cells and Tissue Engineering Unit, Faculty of Oral and Dental Medicine, Cairo University, Giza 12613, Egypt

**Keywords:** stem cells, toll-like receptor, periodontal ligament, polymerase chain reaction, periodontitis, inflammation

## Abstract

Toll-like receptors (TLRs) play a crucial role in the innate immune response, mediating cellular interactions with the microenvironment and influencing periodontal disease progression. This in vitro study aimed to comprehensively characterize the TLR expression profile of periodontal ligament mesenchymal stem/progenitor cells (PDLSCs) and investigate its modulation by inflammatory stimuli associated with periodontal disease. PDLSCs (*n* = 6) were isolated, selected using anti-STRO-1 antibodies, and cultured to evaluate their colony-forming abilities and stem/progenitor characteristics. Baseline and inflammation-induced TLR expressions were evaluated using RT-PCR and protein analyses following cytokine-mediated stimulation. PDLSCs exhibited the expected stem cell characteristics and expressed multiple TLRs under both conditions. Notably, inflammatory stimulation significantly upregulated TLR1 and TLR2 while downregulating TLR10 (*p* < 0.05). These findings provide a comprehensive characterization of TLR expression in PDLSCs and demonstrate how inflammation modulates their innate immune profile. The observed shifts in TLR expression may influence PDLSC responses to microbial pathogens and impact their immunomodulatory and regenerative properties in periodontal tissues. Understanding these interactions could contribute to developing targeted strategies for improving PDLSC-based therapies in periodontal disease.

## 1. Introduction

The periodontal tissues, vital for supporting and anchoring teeth within the alveolar bone, are frequently subjected to various immunological challenges [1]. Periodontal disease is initiated by a dysbiotic microbial biofilm that triggers an exaggerated host immune response, leading to persistent inflammation and progressive destruction of the periodontium [1]. The inflammatory microenvironment in periodontitis is characterized by elevated levels of cytokines, including interleukin-1β (IL-1β), tumor necrosis factor-alpha (TNF-α), and interferon-gamma (IFN-γ), which contribute to the breakdown of the extracellular matrix and alveolar bone loss [2]. This pathological process disrupts tissue homeostasis and impairs the natural regenerative capacity of periodontal structures [2].

In this context, periodontal ligament mesenchymal stem/progenitor cells (PDLSCs) are key cellular players, actively interacting with the surrounding inflammatory microenvironment to regulate immune responses and promote tissue repair [3]. PDLSCs possess unique immunomodulatory properties that allow them to modulate both innate and adaptive immune mechanisms through interactions with inflammatory cytokines, immune cells, and pathogen-associated molecular patterns (PAMPs) [3]. Under physiological conditions, PDLSCs contribute to periodontal homeostasis by differentiating into fibroblasts, osteoblasts, and cementoblasts, supporting the structural integrity of the periodontium [3]. However, inflammatory stimuli can significantly alter PDLSC behavior, affecting their ability to self-renew, differentiate, and exert immunosuppressive functions [4]. This interaction between inflammation and PDLSC-mediated regeneration plays a crucial role in determining the progression or resolution of periodontal disease [4].

Recent studies further emphasize the significant role of PDLSCs in driving various molecular and biological processes at sites of tissue damage or regeneration [4,5,6]. PDLSCs not only contribute to tissue remodeling but also interact with immune cells such as macrophages and T cells [7], influencing the balance between pro-inflammatory and anti-inflammatory responses [4]. Their ability to regulate immune signaling pathways suggests that PDLSCs may serve as potential therapeutic targets for modulating periodontal inflammation and enhancing regenerative outcomes [8].

Toll-like receptors (TLRs) serve as crucial links between the innate and adaptive immune responses by detecting pathogen-associated molecular patterns (PAMPs) and damage-associated molecular patterns (DAMPs) [9]. Ten functional TLRs, both extracellular and intracellular, have been identified and characterized in humans [10]. These receptors exhibit significant immune-modulatory and regenerative capabilities, as TLR activation can influence cellular stemness, differentiation, and immune responses within the local microenvironment [11,12]. While specific TLR expression profiles have been documented for various mesenchymal stem cells derived from the oral cavity, highlighting distinct differences based on their tissue of origin [11,13,14,15,16], a comprehensive TLR expression profile for periodontal ligament stem cells (PDLSCs) has not yet been established [17]. Furthermore, there is a lack of information regarding how inflammation, particularly in the context of periodontal disease, influences TLR expression in these cells. This study aims to characterize, for the first time, the complete TLR expression profile of PDLSCs and to investigate how a standardized cytokine-mediated microinflammatory condition, associated with periodontal disease, could modulate this profile.

## 2. Material and Methods

### 2.1. Isolation and Culture of PDLSCs

The isolation and culture of periodontal ligament mesenchymal stem/progenitor cells (PDLSCs) were conducted in alignment with established protocols for oral mesenchymal stem cells (MSCs) using the tissue explant method [18]. Third molars from six individuals (age in years and gender: 19♂, 19♀, 21♂, 22♂, 23♂, 35♀) were surgically extracted at the community practice of Dr. Kerscher, Dr. Körner, and Föge in Kiel (Ethical Committee IRB-Approval D513/17). Following extraction, the teeth were immediately transferred into sterile 50 mL tubes (Sarstedt AG, Nümbrecht, Germany) containing 15 mL of the basic medium. This medium consisted of Minimum Essential Medium Eagle Alpha Modification (α-MEM, Sigma-Aldrich GmbH, Hamburg, Germany) supplemented with antibiotics (100 U/mL penicillin, 100 µg/mL streptomycin), 1% amphotericin, 400 mmol/mL L-glutamine (all from Biochrom AG, Berlin, Germany) and 15% fetal calf serum (FCS, HyClone, Logan, UT, USA).

Subsequent processing took place in the cell culture laboratory of the Clinic for Conservative Dentistry and Periodontology of Kiel University under a safety cabinet (Hera Safe, Thermo Fisher Scientific, Waltham, MA, USA). The basal medium was removed (Vacusafe Comfort, IBS Integra Biosciences, Chur, Switzerland), and the teeth were washed three times with phosphate-buffered saline (PBS) (Biochrom), briefly disinfected with 70% ethanol, and washed again with PBS. The periodontal ligament tissue was carefully dissected from the tooth root using sterile instruments into approximately 3 mm pieces and allowed to adhere to the bottom of dry 75 mL culture flasks (Sarstedt AG, Nümbrecht, Germany) for 30 min. The samples were then covered with the basic medium and incubated undisturbed at 37 °C with 5% CO_2_ for one week in an incubator (Binder GmbH, Tuttlingen, Germany), and the medium was renewed thrice a week. When the cells reached 80% confluence, they were passaged. Cells were separated using magnetically activated cell sorting (MACS), which involved attaching STRO-1 surface markers to anti-STRO-1 antibodies and anti-IgM Micro Beads (Table 1) according to the manufacturers’ protocols. MACS positively sorted cells, identified as periodontal mesenchymal stem/progenitor cells (PDLSCs) were expanded in culture and utilized up to passage 5 to ensure cellular viability and consistency. For all experiments, individual cell lines were maintained separately to preserve their distinct biological characteristics and avoid cross-contamination.

### 2.2. Colony-Forming Units (CFUs)

PDLSCs were seeded at a density of 1.63 cells/cm^2^ and cultured under standard conditions. A colony was identified as a cluster containing 50 or more cells. After 12 days, representative samples were fixed with 4% formalin and stained with 0.1% crystal violet. The remaining PDLSCs that formed CFUs were individually isolated using cell scrapers, transferred to new 75 mL flasks, and further cultured under standard conditions.

### 2.3. Flow Cytometric Analysis of MSCs Surface Markers

When PDLSCs reached 80% confluence, their predefined surface markers were analyzed using flow cytometry, following the guidelines established by Dominici et al. [19] and similar to previous investigations [20,21]. Standard procedures were employed, including the use of FcR Blocking Reagent (Miltenyi Biotec) to prevent non-specific Fc receptor binding, ensuring accurate antibody-target interaction. The cells were incubated with fluorochrome-conjugated monoclonal antibodies, including CD73, CD90, and CD105 as positive MSC markers and CD14, CD34, and CD45 as negative markers (Table 1). Corresponding isotype controls were used to establish the baseline fluorescence and exclude non-specific binding (Table 1).

The stained cells were analyzed using the FACSCalibur E6370 system (Becton Dickinson, Heidelberg, Germany), and data acquisition was performed with FACSComp 5.1.1 software. The gating strategy was implemented to ensure accurate and reproducible results. Initially, forward scatter (FSC) and side scatter (SSC) parameters were used to gate viable cells while excluding debris and dead cells. To further refine the analysis, 7-AAD was applied as a live/dead stain, ensuring that only live cells were included. Single-cell gating was then performed by plotting FSC height (FSC-H) against FSC area (FSC-A), thereby eliminating doublets and cell aggregates.

Following the selection of viable single cells, isotype control staining was used to set the fluorescence threshold and define the negative population for each marker. Fluorescence intensity shifts were measured to confirm the positive expression of MSC markers. Cells expressing CD73, CD90, and CD105 were gated as MSC-like populations, whereas cells displaying CD14, CD34, and CD45 were considered hematopoietic and excluded.

### 2.4. Multilineage Differentiation of PDLSCs

To evaluate the osteogenic differentiation potential, 2 × 10^4^ third passage PDLSCs were plated on 6-well culture plates and cultured in an osteogenic induction medium (PromoCell, Heidelberg, Germany). For comparison, identical samples were simultaneously cultured in a basic medium. After two weeks, calcified deposits were stained with Alizarin Red (Sigma-Aldrich), and the expression of runt-related transcription factor 2 (RUNX) and alkaline phosphatase (ALP) and Osteonectin (SPARC) was assessed using real-time polymerase chain reaction (PCR; LightCycler; Roche Molecular Biochemicals, Indianapolis, IN, USA). For adipogenic differentiation, 3 × 10^5^ third passage PDLSCs were cultured on 6-well plates in an adipogenic induction medium (PromoCell), with control samples maintained in a basic medium. Lipid droplet accumulation was detected using Oil Red O staining (Sigma-Aldrich), and the expression of peroxisome proliferator-activated receptor gamma (PPARγ) and lipoprotein lipase (LPL) was measured using PCR after 21 days. Chondrogenic differentiation was initiated by culturing micro-masses of 3 × 10^4^ third passage PDLSCs in a chondrogenic induction medium (PromoCell) on 6-well plates (Sarstedt). Control samples were cultured in a basic medium. On day 35, cells were stained with Alcian Blue (Sigma-Aldrich) to visualize the produced glycosaminoglycans, and aggrecan (ACAN) mRNA expression, a cartilage-specific proteoglycan core protein, was analyzed. The induction medium was refreshed three times a week. All primers were provided by Roche (Table 2).

### 2.5. Inflammatory Medium

To analyze the TLR expression profile of PDLSCs in a standardized inflammatory microenvironment, the cells were exposed to the basic medium supplemented with 25 ng/mL IL-1β, 10^3^ U/mL IFN-γ, 50 ng/mL TNF-α, and 3 × 10^3^ U/mL IFN-α (PeproTech, Hamburg, Germany) for 18 h as previously described [20,22] (PDLSCs-i). The control group received only the basic medium (PDLSCs).

### 2.6. TLR Expression at Gene Level

To evaluate the gene expression levels of TLRs in PDLSCs and PDLSCs-i, messenger RNA (mRNA) was extracted from PDLSCs for each patient in both examined groups using the RNeasy kit (Qiagen, Hilden, Germany). Complementary DNA (cDNA) was synthesized from RNA (1 μg/μL) through reverse transcription, utilizing the QuantiTect Reverse Transcription Kit (Qiagen, Hilden, Germany) in accordance with the manufacturer’s instructions. Real-time polymerase chain reaction (PCR) was conducted using a LightCycler 96 system (Roche Diagnostics, Mannheim, Germany) in a total reaction volume of 20 μL, which included 4 pmol TLR primer, 10 μL Fast Start Essential DNA Probes Master (Roche Diagnostics, Mannheim, Germany), and 5 μL sample cDNA. Real-Time Ready Assays (Table 2) were employed following the manufacturer’s guidelines. Phosphoglycerate kinase 1 (PGK1) served as the reference gene, based on previous studies [23], which demonstrated its stable expression by testing 19 candidate reference genes in both stimulated and unstimulated gingival MSCs using NormFinder analysis. Relative gene expression was quantified using the 2^−ΔΔCt^ method, where ΔCt is defined as Ct (target gene)—Ct (PGK1), as described in previous studies [21,23,24]. All experiments were performed in triplicate and the results were averaged.

### 2.7. TLR Expression at Protein Level

PDLSCs and PDLSCs-i were analyzed for their protein-level expression of TLRs 1–10, using flow cytometry similar to previous investigations [20,21,25]. The cells were fixed and permeabilized using Cytofix/Cytoperm (BD Biosciences, Franklin Lakes, NJ, USA). Viability staining was conducted with VioBlue (Miltenyi Biotec). The binding of primary antibodies and their isotype controls (all listed in Table 1) was performed using 1% FCS. Consequently, results were evaluated with FACSCalibur E6370 and FACSComp 5.1.1 software (Becton Dickinson, Heidelberg, Germany).

### 2.8. Null Hypothesis

This study was conducted under the null hypothesis (H_0_) that inflammatory stimulation does not significantly alter the expression of TLRs in PDLSCs at the mRNA and protein levels. The alternative hypothesis (H_1_) proposed that inflammatory conditions would lead to significant modulation of TLR expression, reflecting the adaptive immune responses of PDLSCs in an inflamed periodontal microenvironment.

### 2.9. Statistical Analysis

The Kolmogorov–Smirnov Test was performed to test for the normality of the data. Data were not normally distributed. Differences between PDLSCs and PDLSCs-i were evaluated using the non-parametric Wilcoxon Signed Rank test using SPSS software (Version 28.0, IBM Corporation, Armonk, NY, USA). The level of significance was set at *p* = 0.05.

## 3. Results

### 3.1. Phase Contrast Inverted Microscopy and Colony Forming Units

**Figure 1:**
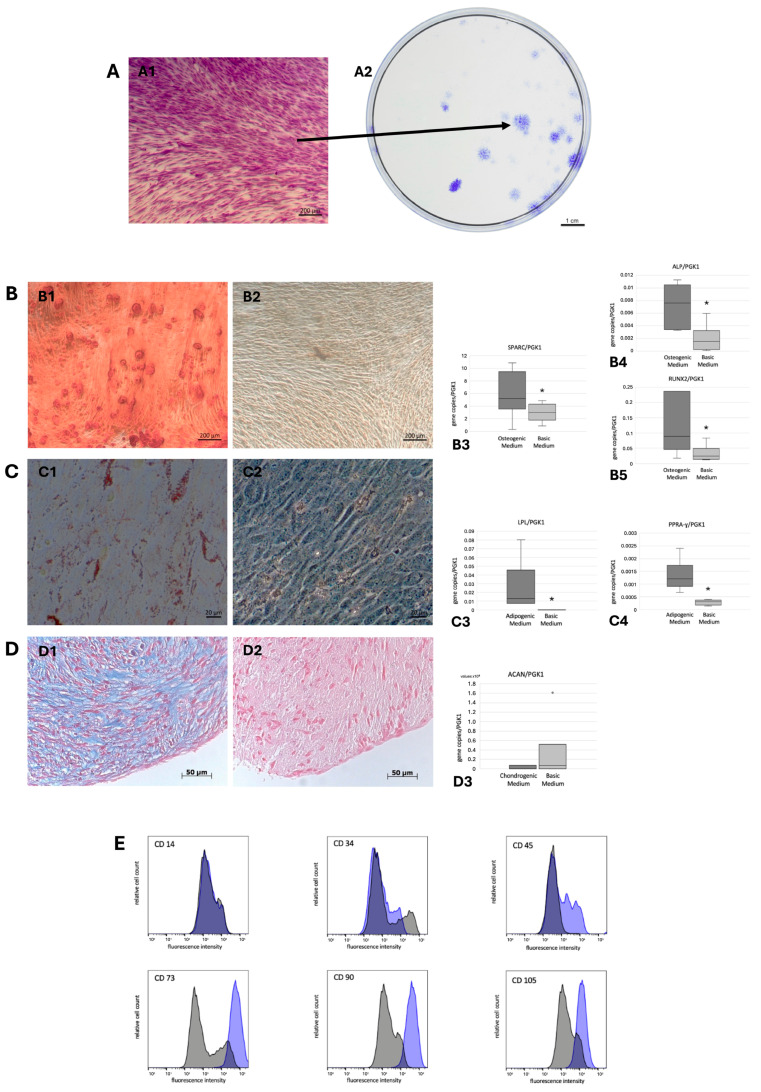
(**A**). Crystal violet staining of PDLSCs‘ colonies formed after 12 days and their (**A1**) microscopic and (**A2**) macroscopic appearance. (**B**–**D**). PDLSCs stimulated with osteogenic inductive medium stained with Alizarin Red (**B1**) and their controls (**B2**) with the analysis of SPARC (**B3**), ALP (**B4**), and RUNX2 (**B5**) expression. PDLSCs stimulated with adipogenic differentiation medium stained with Oil Red O (**C1**) and their controls (**C2**) with the analysis of LPL (**C3**) and PPARɣ (**C4**) expression. PDLSCs stimulated with chondrogenic differentiation medium stained with Alcian Blue and Nuclear Fast Red (**D1**) and their controls (**D2**) with the analysis of ACAN (**D3**). (Box-and-whisker plots with medians and quartiles; Wilcoxon signed rank test, statistical significance marked with asterisk, * *p* < 0.05). ACAN, aggrecan; ALP, alkaline phosphatase; LPL, lipoproteinlipase; PPARɣ, peroxisome proliferator-activated receptor-gamma; RUNX, Runt-related transcription factor-2, SPARC, Osteonectin. (**E**) Flow cytometric analysis of the surface marker expression profile of PDLSCs.

Following the initial adherence of the periodontal ligament soft tissue masses, cells began to proliferate out of them, forming adherent fibroblast-like clusters. By the twelfth day, PDLSCs demonstrated colony-forming units (CFUs; Figure 1A).

### 3.2. Multilineage Differentiation Potential and PDLSCs Characterization

PDLSCs stimulated with osteogenic differentiation medium exhibited calcified deposits, as indicated by Alizarin Red staining, in contrast to control samples. These cells also showed significantly higher expression levels (Median gene copies/PGK1 copies, Q25/Q75) of RUNX (0.0894, 0.0459/0.2365) and ALP (0.0076, 0.0034/0.0105) and SPARC (5.217, 3.546/9.464) compared to controls (RUNX: 0.0246, 0.0139/0.0486; ALP: 0.0015, 0.0003/0.0032; SPARC: 2.950, 1.778/4.320) (Figure 1B). In the adipogenic differentiation assay, PDLSCs formed lipid droplets, which were positively stained with Oil Red O, unlike the control samples, and exhibited significantly higher expression of PPARγ (0.0012, 0.0009/0.0017) and LPL (0.0128, 0.0076/0.0457) compared to controls (PPARγ: 0.0003, 0.0002/0.0004; LPL: 0.0000, 0.0000/0.0001) (Figure 1C). Chondrogenic differentiation led to the production of glycosaminoglycans in PDLSCs, positively stained with Alcian Blue, in contrast to controls. The expression of ACAN (0.0000, 00/7703793) was higher than that of control samples (0.0000, 0.0000/5154252) (Figure 1D). PDLSCs were CD14^−^, CD34^−^, CD45^−^, CD73^+^, CD90^+^, and CD105^+^ (Figure 1E).

### 3.3. TLR Expression in PDLSCs and PDLSCs-i

**Figure 2:**
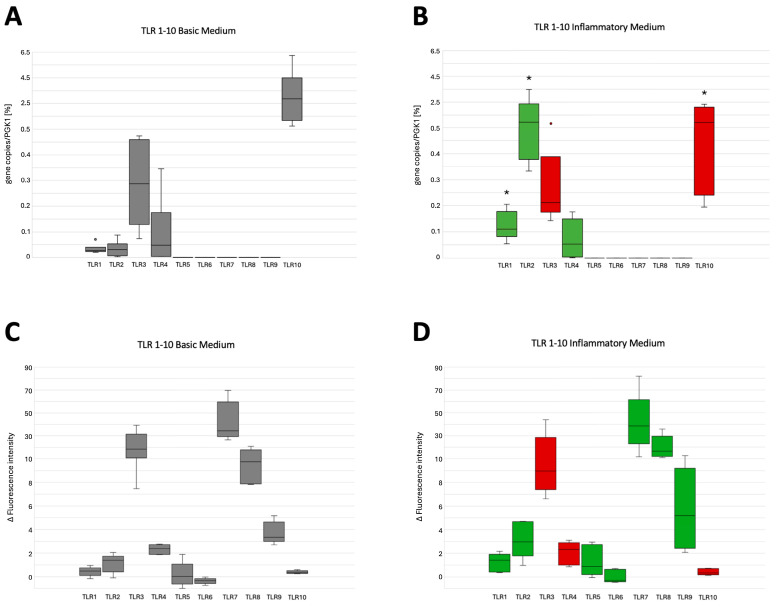
Median gene copies/PKG1 of expressed TLRs in PDLSCs in (**A**) basic and (**B**) inflammatory medium and median fluorescence intensity of PDLSCs in (**C**) basic and (**D**) inflammatory medium. Red boxes indicate a decrease in expression, while green boxes indicate an increase in expression within the inflammatory milieu (Box-and-whisker plots with medians and quartiles; Wilcoxon signed rank test; outliers marked with circle; statistical significance marked with asterisk, * *p* < 0.05).

PDLSCs and PDLSC-i exhibited different expressions of TLRs on the gene level (Median TLR gene copies/PGK1 copies, Q25/Q75) as shown in (Figure 2), with significant upregulation in TLRs 1 and 2 and a significant downregulation in TLR10 (*p* < 0.05). At the protein level, flow cytometric analysis revealed TLR expressions (Median Δfluorescence intensity, Q25/Q75) in both PDLSCs and PDLSC-i, as presented in (Figure 2), with no significant differences observed between the two groups.

## 4. Discussion

Periodontitis is a chronic inflammatory condition that affects the periodontal ligament, gingiva, and alveolar bone [26]. It is primarily initiated by microbial dysbiosis, wherein the oral microbiome shifts towards a pathogenic bacterial composition, leading to an exaggerated host immune response [12]. This prolonged immune activation results in the progressive destruction of the periodontium and, if left untreated, ultimately leads to tooth loss [1]. As a global health concern, periodontitis not only contributes to oral morbidity but has also been strongly associated with systemic conditions such as cardiovascular diseases, diabetes, and adverse pregnancy outcomes [27,28]. The periodontium harbors various mesenchymal stem cell (MSC) populations, including periodontal ligament stem/progenitor cells (PDLSCs), gingival mesenchymal stem cells (G-MSCs), and alveolar bone mesenchymal stem cells (AB-MSCs) [29]. These cells play a critical role in maintaining periodontal tissue homeostasis and facilitating tissue repair following inflammation and injury [30]. Their regenerative capabilities enable differentiation into essential periodontal cell types—osteoblasts, cementoblasts, and fibroblasts—necessary for tissue restoration [31]. Additionally, these MSCs are integral in modulating immune responses to limit inflammatory damage and promote healing [32]. Recent studies have underscored the potential of targeting MSC populations in regenerative approaches for periodontitis, reinforcing the notion that successful therapeutic strategies must address both microbial dysbiosis and stem cell-mediated regeneration to achieve effective and long-lasting periodontal healing [33,34]. Toll-like receptors (TLRs) are essential modulators of MSC function, playing key roles in pathogen recognition and immune responses within inflamed periodontal tissues [35]. Certain TLRs have been identified as regulators of MSC processes, including differentiation, migration, and immunomodulation—functions vital for tissue repair in inflammatory environments [21,36]. This study is the first to comprehensively characterize the TLR expression profile of PDLSCs under both inflamed and non-inflamed conditions. PDLSCs were isolated using anti-STRO-1 antibodies and exhibited typical MSC markers, expressing CD90, CD105, and CD73, while lacking CD14, CD34, and CD45. Their regenerative potential was confirmed through colony formation, multilineage differentiation, and adherence to culture plastic. To simulate the complex inflammatory microenvironment seen in periodontal disease, PDLSCs were exposed to a standardized cytokine cocktail [20].

At the mRNA level, PDLSCs expressed TLRs 1, 2, 3, 4, and 10 under baseline conditions, with TLR10 and TLR3 exhibiting the highest expression. Following inflammatory cytokine stimulation, TLR1, TLR2, and TLR4 were significantly upregulated, whereas TLR3 and TLR10 were downregulated. At the protein level, all ten TLRs were expressed in PDLSCs, with TLR7 and TLR3 exhibiting high expression. The discrepancy between mRNA and protein levels is consistent with known cellular regulatory mechanisms, including post-transcriptional modifications, mRNA stability, and protein degradation [37,38]. These factors contribute to differential protein expression, influencing the persistence and functional impact of TLRs.

Under inflammatory conditions, the protein levels of TLR1, TLR2, TLR5, TLR6, TLR7, TLR8, and TLR9 increased, whereas TLR3, TLR4, and TLR10 levels decreased. Although these changes were not statistically significant, they suggest that TLR protein expression dynamics may require extended exposure to inflammation for substantial modulation [39] or may be regulated by intricate post-translational mechanisms [40]. Comparisons between PDLSCs and other MSCs (Table 3) indicate that certain TLRs, such as TLR1, TLR2, and TLR4, are consistently expressed across multiple MSC populations in the oral cavity and function as primary bacterial sensors, detecting key periodontal pathogens like *Porphyromonas gingivalis* and *Aggregatibacter actinomycetemcomitans* [41,42]. The upregulation of these TLRs reinforces their role in amplifying immune responses during early-stage periodontitis [42]. Interestingly, PDLSCs exhibited uniquely high baseline expression of TLR3 and TLR10. TLR3, which detects viral RNA, has been implicated in regulating MSC function by suppressing inflammatory mediators and promoting osteogenic differentiation, thereby facilitating tissue maturation and regeneration, as seen in G-MSCs [21]. Conversely, TLR10 remains poorly understood but is suggested to have an anti-inflammatory role [43,44], potentially acting as a regulatory modulator to maintain periodontal tissue homeostasis [43,44]. The observed downregulation of TLR3 and TLR10 in inflamed PDLSCs suggests a shift from anti-inflammatory functions of the cells to a bacterially driven pro-inflammatory phenotype, which is characteristic of periodontal disease progression [45]. This aligns with findings that chronic TLR activation by bacterial PAMPs sustains cytokine release, exacerbating tissue destruction and disease progression [46]. Despite the significant mRNA downregulation of TLR3 and TLR10, their proteins remained detectable, suggesting possible post-transcriptional regulatory mechanisms [38] that enable PDLSCs to flexibly adapt their inflammatory responses.

These findings emphasize the complex interplay of TLR signaling in PDLSCs in different stages of periodontitis. The upregulation of TLR1 and TLR2 in inflamed PDLSCs enhances periodontal pathogen recognition and immune activation in the initial stages of the disease [41,42], but their sustained expression may contribute to chronic inflammation through prolonged cytokine production [46], NF-κB [47] signaling, and the inhibition of osteogenesis [48], leading to bone resorption and periodontal destruction [46]. In addition, the downregulation of TLR3 and TLR10 in inflamed PDLSCs suggests a loss of its proposed anti-inflammatory function, which may otherwise counteract excessive immune activation [21,43]. This reduced expression could contribute to heightened inflammation and further periodontal breakdown, as well as potential systemic complications in the later stages of the disease [49]. These observations suggest that targeting TLR pathways, either by inhibiting TLR-mediated inflammation or enhancing TLRs‘ anti-inflammatory activity, could offer promising therapeutic approaches for periodontitis management.

Given the detrimental effects of chronic PDLSCs’ TLR1 and TLR2 activation in an inflammatory milieu, selective inhibition or modulation of these receptors, as well as other periodontitis-associated TLRs, such as TLR4 [50], may help prevent excessive inflammation while preserving necessary antimicrobial defense mechanisms. TLR antagonists or small-molecule inhibitors that downregulate TLR1/2 and TLR4 activity could reduce pro-inflammatory cytokine production, mitigating inflammation-induced bone loss and restoring immune balance [51]. Additionally, this inhibition has been demonstrated to enhance osteogenic differentiation of PDLSCs under inflammatory conditions, thereby promoting periodontal regeneration [52,53,54]. Another strategy could be the modulation of downstream signaling pathways, such as MAPK or NF-κB [47,53,55], which could further regulate hyperinflammatory responses while maintaining appropriate pathogen recognition.

TLR3 activation may also provide regenerative potential in periodontitis. Studies indicate that synthetic agonists like Poly(I:C) can stimulate TLR3 in MSCs, enhancing anti-inflammatory properties and promoting tissue repair [21]. Pharmacological strategies that reinforce TLR3 expression in PDLSCs could counteract its downregulation during inflammation and further enhance tissue regeneration. This could potentially be achieved by incorporating natural bioactive compounds, such as thymoquinone [24,56] and polyphenols [57], as adjunctive periodontal therapies, as suggested by other investigations. Combining these molecular approaches with conventional periodontal treatments, such as professional mechanical plaque removal and novel adjunctive therapies like platelet derivatives [58,59], may provide a comprehensive strategy that not only controls inflammation but also supports periodontal regeneration.

Furthermore, enhancing TLR10 function could restore immune-regulatory balance [44], reducing tissue-destructive inflammation and promoting long-term periodontal stability. Future research should explore therapeutic strategies to activate or mimic TLR10 function, investigating its role as a key modulator in preventing immune hyperresponsiveness in periodontitis. As periodontal research advances, a deeper understanding of TLR-mediated immune responses will be crucial in developing targeted therapies that effectively control inflammation while fostering tissue repair and long-term periodontal health.

Yet, although this study offers insights into TLR expression in PDLSCs under inflammatory and non-inflammatory microenvironment conditions, several limitations are noteworthy. First, inflammation was modeled using a standardized short-term cytokine exposure, which may not capture the chronic nature of periodontitis. Since periodontitis is a prolonged condition, long-term inflammatory effects on TLR expression and PDLSC function, including potential adaptive responses or cell exhaustion, were not addressed here. Additionally, while in vitro models provide valuable mechanistic insights, the expression of TLRs in cultured PDLSCs may not fully reflect their in vivo behavior. In the periodontal niche, PDLSCs interact dynamically with immune cells, extracellular matrix components, and microbial biofilms, all of which contribute to TLR signaling and modulation . These interactions introduce regulatory mechanisms that are absent in isolated cell cultures, potentially influencing TLR expression in a manner not replicated in vitro. Moreover, external factors such as oxygen tension [60], mechanical loading [61], and cytokine gradients [62] play a role in modulating TLR expression and were not accounted for in this study. Another key limitation is the absence of continuous microbial stimulation, a defining feature of the periodontal environment [63]. The oral cavity is constantly exposed to bacterial ligands that shape TLR responses, affecting immune activation and tissue homeostasis [35]. The lack of such microbial interactions in our in vitro model may lead to differences in TLR regulation compared to natural conditions, emphasizing the need for translational models that more closely mimic the in vivo periodontal setting. Despite these limitations, PDLSC cultures remain a powerful tool for dissecting molecular mechanisms underlying TLR regulation. However, to bridge the gap between in vitro observations and clinical relevance, future studies should consider 3D tissue-engineered constructs or co-culture models that integrate microbial and immune system interactions. Such approaches will provide a more comprehensive understanding of TLR-mediated immune responses in periodontitis and enhance the translational potential of PDLSC-based therapies.

## 5. Conclusions

This study identifies distinct TLR expression patterns in PDLSCs in basic and inflammatory microenvironments, advocating a key role of TLRs in immune regulation and repair/regeneration within the process of periodontal disease. Future research on specific TLR modulation may advance further understanding of periodontal disease processes with potential therapeutic strategies to enhance periodontal healing and inflammatory response modulation in periodontitis.

## Figures and Tables

**Table 1:** Detailed list of antibodies and isotype controls showing manufacturers, fluorophore labels, and catalog numbers.

Antibody or Isotype Control	Manufacturer	Catalog Number
AF488 conj. CTRL	Santa Cruz Biotechnology, Dallas, TX, USA	sc-3890
Anti PE Micro Beads	Miltenyi Biotec, Bergisch Gladbach, Germany	139-048-801
Anti TLR1 PE	eBioscience, Thermo Fisher Scientific, Waltham, MA, USA	12-9911-41
Anti TLR2 FITC	BioLegend, San Diego, CA, USA	309705
Anti TLR3 PE	eBioscience, Thermo Fisher Scientific, Waltham, MA, USA	12-9039-80
Anti TLR4 FITC	Enzo Life Sciences, Farmingdale, NY, USA	ALX-804-419FT100
Anti TLR5 AF488	R&D, Minneapolis, MN, USA	FAB6704G
Anti TLR6 PE	BioLegend, San Diego, CA, USA	334707
Anti TLR7 PE	eBioscience, Thermo Fisher Scientific, Waltham, MA, USA	MA5-16249
Anti TLR8 PE	Enzo Life Sciences, Farmingdale, NY, USA	ALX-804-376R-C100
Anti TLR9 PE	eBioscience, Thermo Fisher Scientific, Waltham, MA, USA	12-9099-82
Anti TLR10 PE	eBioscience, Thermo Fisher Scientific, Waltham, MA, USA	12-2909-42
CD105 FITC	Miltenyi Biotec, Bergisch Gladbach, Germany	130-112-327
CD14 FITC	Miltenyi Biotec, Bergisch Gladbach, Germany	130-110-576
CD146 PE	Miltenyi Biotec, Bergisch Gladbach, Germany	130-097-939
CD34 PE	Miltenyi Biotec, Bergisch Gladbach, Germany	130-113-741
CD45 APC	Miltenyi Biotec, Bergisch Gladbach, Germany	130-110-771
CD73 PE	Miltenyi Biotec, Bergisch Gladbach, Germany	130-112-060
CD90 FITC	Miltenyi Biotec, Bergisch Gladbach, Germany	130-114-901
IgG1 PE	BD Biosciences, Franklin Lakes, NJ, USA	349043
IgG2a APC	Miltenyi Biotec, Bergisch Gladbach, Germany	130-091-836
IgG2a PE	BD Biosciences, Franklin Lakes, NJ, USA	349053
IgM PE	Santa Cruz Biotechnology, Dallas, TX, USA	sc-2870
Mouse IgG1κ PE CTRL	BioLegend, San Diego, CA, USA	400112
Mouse IgG1 PE	BD Biosciences, Franklin Lakes, NJ, USA	345816
Mouse IgG2a FITC	BD Biosciences, Franklin Lakes, NJ, USA	349051
Mouse IgG2a κ PE	BioLegend, San Diego, CA, USA	400213
Rat IgG1k PE	eBioscience, Thermo Fisher Scientific, Waltham, MA, USA	12-4301-82
REA Control FITC	Miltenyi Biotec, Bergisch Gladbach, Germany	130-113-437
REA Control PE	Miltenyi Biotec, Bergisch Gladbach, Germany	130-113-438
STRO-1 PE	Santa Cruz Biotechnology, Dallas, TX, USA	sc-47733

**Table 2:** Primers used for evaluating multilineage differentiation and Toll-like receptor expression.

Gene-Symbol	Assay-ID	Accession-ID	Species	Description
PGK1	102083	ENST00000373316	*H. sapiens*	Phosphoglycerate kinase 1
ALP	103448	ENST00000374840	*H. sapiens*	Alkaline phosphatase
RUNX2	113380	ENST00000359524	*H. sapiens*	Runt-related transcription factor 2
SPARC	103218	ENST00000231061	*H. sapiens*	Osteonectin
LPL	113230	ENST00000311322	*H. sapiens*	Lipoprotein lipase
PPARɣ	110607	ENST00000287820	*H. sapiens*	Peroxisome proliferator-activated receptor gamma
ACAN	138057	ENST00000439576	*H. sapiens*	Aggrecan core protein
TLR1	111000	ENST00000308979	*H. sapiens*	Toll-like receptor 1
TLR2	145617	ENST00000260010	*H. sapiens*	Toll-like receptor 2
TLR3	111008	ENST00000296795	*H. sapiens*	Toll-like receptor 3
TLR4	135752	ENST00000355622	*H. sapiens*	Toll-like receptor 4
TLR5	103674	ENST00000366881	*H. sapiens*	Toll-like receptor 5
TLR6	111018	ENST00000381950	*H. sapiens*	Toll-like receptor 6
TLR7	111012	ENST00000380659	*H. sapiens*	Toll-like receptor 7
TLR8	103816	ENST00000218032	*H. sapiens*	Toll-like receptor 8
TLR9	143252	ENST00000360658	*H. sapiens*	Toll-like receptor 9
TLR10	141065	NM_001017388	*H. sapiens*	Toll-like receptor 10

**Table 3:** Reported TLR expression of oral mesenchymal stem cells in basic and inflammatory conditions.

	TLR	1	2	3	4	5	6	7	8	9	10	References
**PDLSCs**	**b**	**+**	**+**	**+**	**+**	**+**	-	**+**	**+**	**+**	**+**	Current study
	**i**	**+**	**+**	**+**	**+**	**+**	**+**	**+**	**+**	**+**	**+**	
**G-MSCs**	**b**	+	+	+	+	+	+	+	+	+	+	[15,25]
	**i**	+	+	+	+	+	+	+	-	-	+	
**DPSCs**	**b**	+	+	+	+	+	+	+	+	+	+	[14]
	**i**	+	+	+	+	+	+	+	+	+	+	
**SCAP**	**b**	+	+	+	+	+	+	+	+	+	+	[16]
	**i**	+	+	+	+	+	+	+	+	+	+	
**SHEDs**	**b**	+	+	+	+	+	+	+	+	+	+	[20]
	**i**	+	+	+	+	+	+	+	+	+	+	
**AB-MSCs**	**b**	+	+	+	+	+	+	+	+	+	+	[13]

b: basic culture conditions. i: inflammatory conditions or cells derived from inflamed tissue, +: TLR expression detected, -: no TLR expression detected. Abbreviations: MSCs derived from the periodontal ligament (PDLSCs), gingiva (G-MSCs), dental pulp (DPSCs), apical papilla (SCAP), deciduous tooth pulp (SHEDs), alveolar bone (AB-MSCs).

## Data Availability

The data presented in this study are available on request from the corresponding author. Due to ethical and privacy restrictions, certain data may not be publicly accessible.
